# WeChat as a platform for blending problem/case-based learning and paper review methods in undergraduate paediatric orthopaedics internships: a feasibility and effectiveness study

**DOI:** 10.1186/s12909-023-04269-2

**Published:** 2023-05-08

**Authors:** Junfei Chen, Bingjun Gao, Kunyao Wang, Yinghan Lei, Shengling Zhang, Shaobin Jin, Weiwei Yang, Yan Zhuang

**Affiliations:** 1 Department of Paediatric Surgery, Qilu Hospital of Shandong University, Jinan, 250012 Shandong People’s Republic of China; 2 Shandong University, Jinan, 250012 Shandong People’s Republic of China

**Keywords:** WeChat, Problem-based learning, Case-based learning, Paper review method, Online education

## Abstract

**Background:**

Paediatric orthopaedics is a significant and difficult for undergraduate students to master. During the COVID-19 pandemic, we used the WeChat platform to combine the advantages offered by problem-based learning (PBL), case-based learning (CBL) and paper review teaching methods to establish a new blended online teaching model and demonstrated its feasibility and effectiveness.

**Objective:**

This study aims to demonstrate the feasibility and effectiveness of a new blended pedagogical method that uses the WeChat platform and combines PBL, CBL and paper review.

**Methods:**

We enrolled 22 students participating in the Department of Paediatric Orthopaedics. They participated in the WeChat blended pedagogy mode. Their departmental rotation examination scores were compared with those of 23 students who participated in the traditional teaching method. Moreover, an anonymous questionnaire was used to evaluate students’ perceptions and experiences.

**Results:**

The total average scores of students who participated in the WeChat blended pedagogy mode and the traditional teaching method were 47.27 and 44.52, respectively. There were no statistically significant differences between the online teaching mode and the traditional teaching method in terms of possessing professional accomplishment, gaining knowledge and promoting interpersonal skills (*P* = 0.07, *P* = 0.12 and *P* = 0.65, respectively). In terms of independent clinical thinking, self-improving capability and improving clinical skills, the scores associated with the WeChat blended pedagogy mode were 8.00, 8.00 and 6.00, whereas those associated with the traditional teaching method were 6.70, 6.87 and 7.48. The overall satisfaction with the WeChat blended pedagogy mode reached 100%. A total of 64%, 86%, 68%, 64% and 59% of students chose very large or large in response to the items concerning professional accomplishment, knowledge absorption, independent clinical thinking skills, English reading and literature exploring capacity, as well as interpersonal skills, respectively. Fifteen participants claimed that the WeChat blended pedagogy mode was less helpful to them with regard to promoting the improvement of their clinical skills. Nine students claimed that the WeChat blended pedagogy mode was time-consuming.

**Conclusions:**

Our study verified the feasibility and effectiveness of the WeChat blended pedagogy mode for undergraduate paediatric orthopaedics internships.

**Trial Registration:**

Retrospectively registered.

**Supplementary Information:**

The online version contains supplementary material available at 10.1186/s12909-023-04269-2.

## Introduction

In December 2019, a series of cases of a novel type of pneumonia was reported in Wuhan, China. The respective infection was named “coronavirus disease 2019 (COVID-19)” [1]. As of June 2022, more than 600 million cases have been diagnosed and more than 6.5 million people have died from this disease [2]. Because large crowds can greatly increase the infection rate of COVID-19, academic institutions have been shut down, and training and teaching have been put to a halt in many countries, including China [3–5]. Therefore, online teaching has become the main teaching method used in China during the COVID-19 epidemic [6].

WeChat, a mobile phone-based social networking service similar to WhatsApp [7], is one of the fastest-growing mobile apps and the most popular platform visited daily by university students in China [8]. And more importantly, WeChat has powerful functions, including the ability to send messages in various formats (e.g., texts, videos, and images) to an individual or a specific group, check-in, punch-in, examination, assessment and interaction. So WeChat has become increasingly popular as an interactive communication tool used to improve the efficiency of medical education in recent years [9–11].

Paediatric orthopaedics is an important branch of paediatric surgery that explores orthopaedic surgery-related diseases in the context of all children from birth to adulthood, as well as related medical education and basic research. The clinical manifestations of paediatric orthopaedic patients lack typicality and intuitiveness because of unclear presentation and poor positioning. Some acute diseases, such as supracondylar fracture of the humerus and femoral shaft fracture, can cause lifelong disability or death if not treated correctly in a timely manner. Both the diagnosis and treatment of paediatric orthopaedic diseases are complex, which makes it both significant and difficult for undergraduate students to master basic knowledge regarding paediatric orthopaedic diseases.

However, the traditional teaching model has a deep-rooted influence on the majority of faculty, and they continue to employ teacher-centred, class-oriented didactic lectures and exam-oriented course teaching, which causes students to exhibit a passive state of “acceptance” [12]. Therefore, medical undergraduates lack learning initiative and enthusiasm, and they cannot put the knowledge they have learned into practice, which causes them to forget this knowledge more easily. So students must shift from a passive role to an active role to learn the paediatric orthopaedics. The choice of appropriate teaching methods in this context is crucially important.

Problem-based learning (PBL) is a student-centred pedagogy in which students learn by solving listed questions. It guides students to find and solve clinical problems in response to preset questions and gradually to provide diagnosis conditions [13]. Case-based learning (CBL) is defined as a case-based education method that is grounded in the analysis of medical records and aims at restoring the real clinical scene and prompting students to identify and develop new areas of learning [14]. PBL and CBL approaches have been introduced into medical education with great success, leading medical students to obtain significantly higher levels of knowledge and skill and to exhibit excellent academic performance and higher success rates in examinations [13–16]. However, in isolation, neither PBL nor CBL is without limitations [17]. The problems included in PBL teaching sometimes deviate from the clinical practice. Students cannot apply this knowledge to clinical cases [18]. Furthermore, PBL teaching features a great deal of uncertainty regarding the breadth and depth of learning without a syllabus [19], which is not beneficial to maintaining students’ interest in learning. CBL, for its part, demands that teachers create a set of questions for students to discuss, leading to a tendency for students to lack proactive involvement in and general enthusiasm for the learning experience [17]. In addition, PBL or CBL both require participants to spend a great deal of time preparing materials before class, which is extremely difficult for medical undergraduates. Because their literature retrieval ability is weak, their means of acquiring knowledge basically relies on textbook knowledge. As a result, the discussion in the PBL or CBL teaching process cannot fully mobilize students’ subjective initiative.

The paper review teaching mode refers to the process of guiding students to read specified and relevant foreign literature during the teaching process, which can improve students’ ability to read, summarize and refine information, emphasizes the summary and expansion of knowledge, and ultimately allows the student to return to solving clinical practical problems [20]. This teaching mode is a kind of collective reading method with a clear purpose and a strong sense of design, which can greatly compensate for the shortcomings of students’ weak literature retrieval ability.

Therefore, we hypothesize that a new pedagogical mode that is based on the WeChat platform and combines the virtues of PBL, CBL and paper review can achieve the goal of promoting high-quality undergraduate paediatric orthopaedics internship learning more effectively, and we aim to demonstrate the feasibility and effectiveness of this approach.

## Materials and methods

### Participants

The study was conducted by reference to 22 fourth-year students majoring in clinical medicine at the Qilu Medical College of Shandong University who participated in a 2-week internship in the Department of Paediatric Orthopaedics at the Qilu Hospital of Shandong University from November to December 2020. A total of 4 groups were included, and each group comprised 5 or 6 students. Two clinical doctors with more than 3 years of experience teaching paediatric surgery content and more than 1 year of experience teaching online as well as 1 standardized patient were enrolled in this study. The integration of standardized patients has been proven to be a feasible learning strategy [21]. All participants participated in the new WeChat blended pedagogy mode. Informed consent was provided by the participants, and signed consent forms were collected to the beginning of the study. The study was approved by the Medical Council of Qilu Hospital, Shandong University.

### WeChat blended pedagogy mode

The new blended pedagogy mode was constructed on the basis of the WeChat app. WeChat is a popular app that is available on Android, iPhone, and Windows, and is supported by Wi-Fi, 4G, and 5G data networks. Using WeChat, students can communicate with each other anywhere and at any time [22]. All participants had their own mobile phones and were required to install WeChat. All participants were familiar with the practical aspects of WeChat.

We chose supracondylar fractures of the humerus, developmental dysplasia of the hip, polydactyly, acute osteomyelitis and bone cysts as the topics to which the WeChat blended pedagogy mode would be applied in this study. Because these five diseases are common and occur frequently in paediatric orthopedics, the diagnosis and treatment of these diseases are key for students to master in the Department of Paediatric Orthopedics.

During the 2-week internship in paediatric surgery, students are required to learn about the five diseases described above. Each course lasts for 2 days. In the morning of the first day, students follow the teacher’s mobile phone video online through daily ward rounds, and in the afternoon of the first day and the second day, they use the WeChat platform for online teaching.

Every course is introduced by a real clinical case. Clinical cases are presented to students in terms of three parts. The first part includes medical history and simple medical history. Students must ask questions of standardized patients based on their own diagnosis and treatment ideas and obtain improved case information. The second part includes the results of the physical examination, laboratory examination and imaging examination. The third part is the operation picture or video. Each section is followed by 2–3 questions for discussion.

**Fig. 1:**
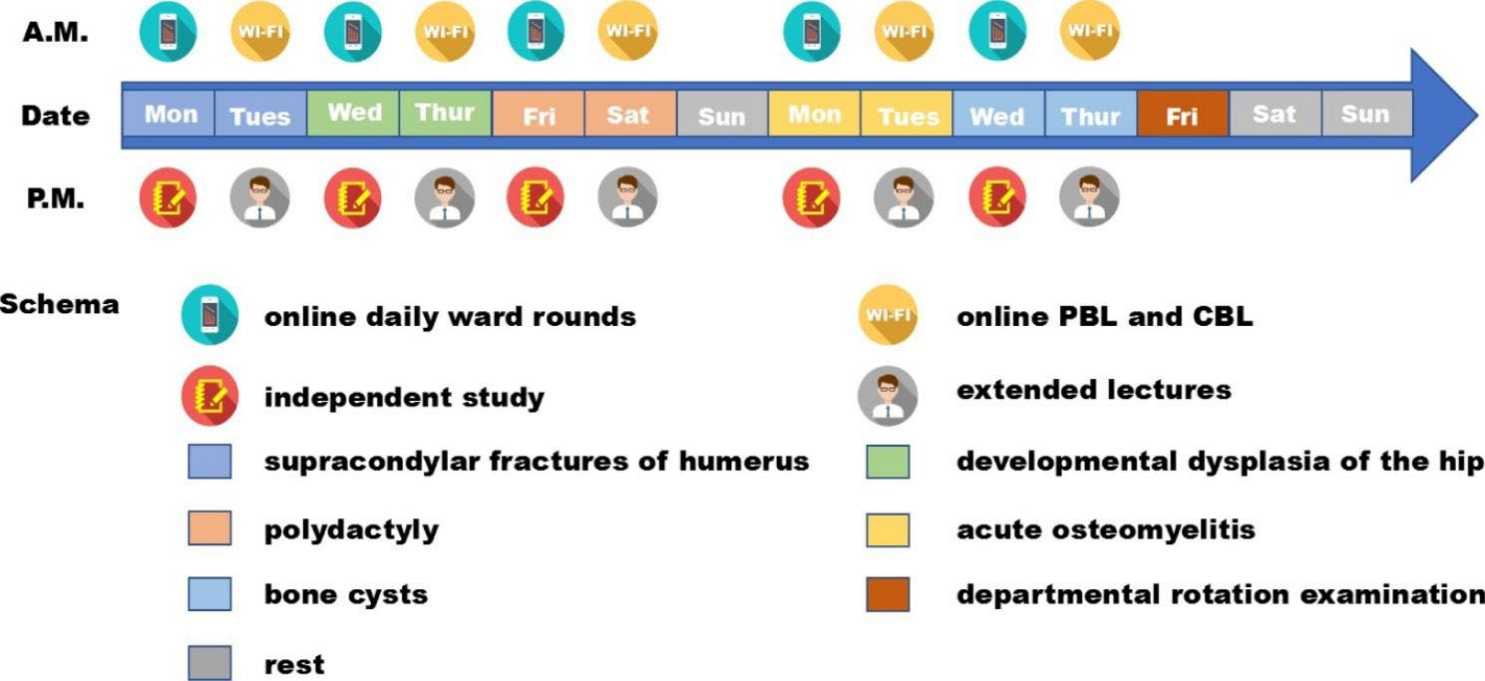
Basic flowchart of the WeChat blended pedagogy mode

The two days of each course are arranged as follows (Fig. 1). In the afternoon of the first day, students mainly learn book knowledge independently, read 2–3 specified foreign relevant literature reviews provided by the teacher, and summarize this knowledge based on questions that have been prepared in advance. In the morning of the subsequent day, based on the first part of clinical cases provided by the teacher, students began to discuss and study in groups, and gradually present the case content alongside the discussion process to complete the teaching process. In the afternoon, the teacher gives extended lectures based on the differential diagnosis of diseases. On the final day of the internship, students completed the departmental rotation examination and the subjective anonymous questionnaire.

### Evaluating the wechat blended pedagogy mode

The key to evaluating the WeChat blended pedagogy mode was to determine whether its teaching effect could be comparable to or exceed that of the offline teaching method. The evaluations included two sections: (1) the departmental rotation examination, which is an objective and comprehensive evaluation provided by the teacher based on student’s performance, and (2) a subjective evaluation of the whole WeChat blended pedagogy mode experience after the 2-week internship.

At the time of the departmental rotation examination, all students were assessed in terms of the same questions. A 2-year-old boy exhibited swelling, pain, and limited mobility after elbow trauma. Based on this chief complaint, the students began to ask questions of the standardized patients. According to the answers of the standardized patients, the students developed their own diagnosis and treatment procedures and briefly described the operation process. The teacher evaluated the students in terms of six aspects based on their performance: possessing professional accomplishment, gaining knowledge, improving clinical skills, developing independent clinical thinking, promoting interpersonal skills and self-improving capability (detailed grading rules are shown in Supplemental Information Table 1). Five grades were used to assess each aspect, i.e., excellent, good, medium, poor and extremely poor, and the difference in score between each grade was 2 points. A maximum score of 10 points were given for each item on a 60-point scale. All the evaluation indicators were based on Bloom’s Taxonomy [23], which categorizes cognitive activities into six hierarchical levels, namely, memory, understanding, application, analytical skills, assessment, and creativity. In November and December 2018, the same questions were also used as part of trainee doctors’ admission tests, which were taken offline because there was no pandemic at that time. These trainee doctors participated in the traditional teaching method, in which the teacher taught knowledge in the classroom and performed ward rounds at the patient’s bedside, and students developed knowledge on their own after the internship.

Subsequently, students were required to complete the same anonymous subjective questionnaire (shown in Supplemental Information Table 2) to evaluate their feedback and perceptions regarding the usage and qualitative utility of the WeChat blended pedagogy mode. The questionnaire consisted of 10 questions, including questions regarding general impressions, doctors’ professional quality, knowledge absorption, improving clinical skills, clinical thinking skills, English reading and literature exploring capacity, interpersonal skills, student-teacher interaction and platform utilization convenience as well as how much free time the course required. The questions were discussed by the 2 clinical doctors who participated in this research to ensure the quality of the questionnaire. Students were asked to estimate the degree to which each item influenced them on a 5-point Likert scale ranging from 1 “strongly dis-influenced” to 5 “strongly influenced”. As in the case of the other scoring areas, with regard to time consumption, grades 1 to 5 represented a scale ranging from a great deal to little. Furthermore, in the WeChat blended pedagogy mode, students’ preparation time was recorded as having been spent previewing the textbook and reading the relevant foreign literature reviews provided by the teacher as well as searching for supplemental materials on the internet. The reliability and validity of the questionnaire was evaluated. The Cronbach’s alpha coefficient was 0.836 and the Kaiser–Meyer–Olkin’s (KMO) test value was 0.684. [17, 24, 25]

### Data analysis

The total scores of the departmental rotation examination were compiled, and the scores of the trainee doctors who participated the WeChat blended pedagogy mode were compared with those of the other 4 groups. In total, 23 trainee doctors who studied in the Department of Paediatric Orthopaedics during the same period in 2018 using the traditional teaching method were included as a reference to evaluate the teaching quality of both online and offline teaching. And their tutors were the same as those in this study. All the students in online and offline teaching have been studying clinical medicine in the School of Medicine of Shandong University for 3 years, but have no clinical practice experience in pediatric surgery. Their school records in systematic anatomy and surgery were taken to assess whether there were differences in their learning abilities and attitudes. The average scores on each section of the rotation examination and previous achievements in systematic anatomy and surgery were calculated by summing all the subitem scores and dividing by the total number of participants involved. Subsequently, the results attained by each of the two groups were compared using an independent sample T test. Measurement data were expressed in terms of the mean ± standard deviation.

Percentages were used to describe the overall results of the anonymous subjective questionnaire and were calculated based on the number of participants who agreed with each item divided by the total number of participants (N = 22).

Data were analysed using statistical package for the social sciences version 23 (SPSS Inc., Chicago, IL) statistical software. *P* values of less than 0.05 were considered to be significant.

## Results

### Basic characteristics and information

A total of 22 fourth-year students participated in an internship in the Department of Paediatric Orthopaedics at the Qilu Hospital of Shandong University from November to December 2020, which employed the WeChat Blended Pedagogy Mode. The mean age of these students was 20.91 ± 0.133. In this sample, the ratio of males to females was 1:1, and these two genders each accounted for 50% of the total. We collected the rotation examination results of paediatric orthopaedic interns in the same period in 2018, which included a total of 4 groups consisting of a total of 23 people. This group used traditional bedside apprenticeship and face-to-face teaching methods. The mean age of these students was 20.82 ± 0.156. The traditional sample included 8 female students, accounting for 34.8% of the total. Table 1 compares the basic characteristics of students who participated in the WeChat blended pedagogy mode and the traditional teaching method. There were no significant differences between the two groups in terms of gender or age (*P* = 0.46 and *P* = 0.66, respectively). In addition, the students’ previous scores in systematic anatomy and surgery in the two groups were shown in Table 1, and there was no statistical difference between them. So there is no difference in their learning ability and attitude.

**Table 1:** The basic characteristics of all the participants

Item	WeChat Blended Pedagogy Mode (N = 22)	Traditional Teaching Method (N = 23)	Statistics	*P* value
**Gender**			χ2 = 0.534	0.46
Male	11 (50%)	15 (65.2%)		
Female	11 (50%)	8 (34.8%)		
**Age**	20.91 ± 0.133	20.82 ± 0.156	T = 0.850	0.66
**Previous achievements**				
Systematic anatomy	81.05 ± 6.86	81.91 ± 6.07	T = -0.449	0.66
Surgery	75.32 ± 6.36	74.65 ± 6.43	T = 0.349	0.73

### Comparison of the departmental rotation examination scores between the wechat blended pedagogy mode and the traditional teaching method

We systematically evaluated the teaching quality associated with the two models in terms of possessing professional accomplishment, gaining knowledge, improving clinical skills, developing independent clinical thinking, and promoting interpersonal skills and self-improving capability; this evaluation was based on the same departmental rotation examination. As illustrated in Table 2, the total average scores of students who participated in the WeChat blended pedagogy mode and the traditional teaching method were 47.27 and 44.52, respectively, a difference which was not statistically significant (*P* = 0.08). This finding showed that the quality of clinical internship teaching using WeChat as a platform for blended problem/case-based learning and paper review methods can be comparable to or even exceed that of offline teaching (total scores 47.27 > 44.52). There were no statistically significant differences between the WeChat blended pedagogy mode and the traditional teaching method in terms of possessing professional accomplishment, gaining knowledge and promoting interpersonal skills (*P* = 0.07, *P* = 0.12 and *P* = 0.65, respectively) (shown in Table 2). In this context, the training of interpersonal skills benefited from the inclusion of standardized patients in online teaching, which *closely* imitated the process of face-to-face consultation with patients in clinical practice; accordingly, the interpersonal skills scores of interns participating in the WeChat blended pedagogy mode in interpersonal skills were no lower than those of interns who participated in the traditional teaching method. However, with regard to the development of independent clinical thinking, the WeChat blended pedagogy mode was obviously superior to the traditional teaching method (average score 8.00 > 6.70, *P* = 0.001), which may be due to the inclusion of problem/case-based learning in the blended teaching method. In addition, in terms of self-improving capability, the scores of interns participating in the WeChat blended pedagogy mode and those of interns participating in the traditional teaching method (total scores 8.00 and 6.87, respectively) indicated that the WeChat blended pedagogy mode had a clear advantage (*P* = 0.01) (shown in Table 2), which was closely related to the use of the paper review teaching mode.

**Table 2:** The scores of the departmental rotation examination of all the participants

Item	WeChat Blending Pedagogy Mode (N = 22)	traditional teaching method (N = 23)	T	*P* value
Possessing Professional Accomplishment	8.64 ± 1.68	7.74 ± 1.51	1.881	0.07
Gaining Knowledge	8.27 ± 1.55	7.57 ± 1.47	1.570	0.12
Improving Clinical Skills	6.00 ± 1.38	7.48 ± 1.50	-3.438	0.001
Developing Independent Clinical Thinking	8.00 ± 1.38	6.70 ± 1.29	3.267	0.002
Promoting Interpersonal Skills	8.36 ± 1.47	8.17 ± 1.34	0.453	0.65
Self-improving Capability	8.00 ± 1.23	6.87 ± 1.58	2.686	0.010
Total average score	47.27 ± 5.91	44.52 ± 4.27	1.784	0.08

**Fig. 2:**
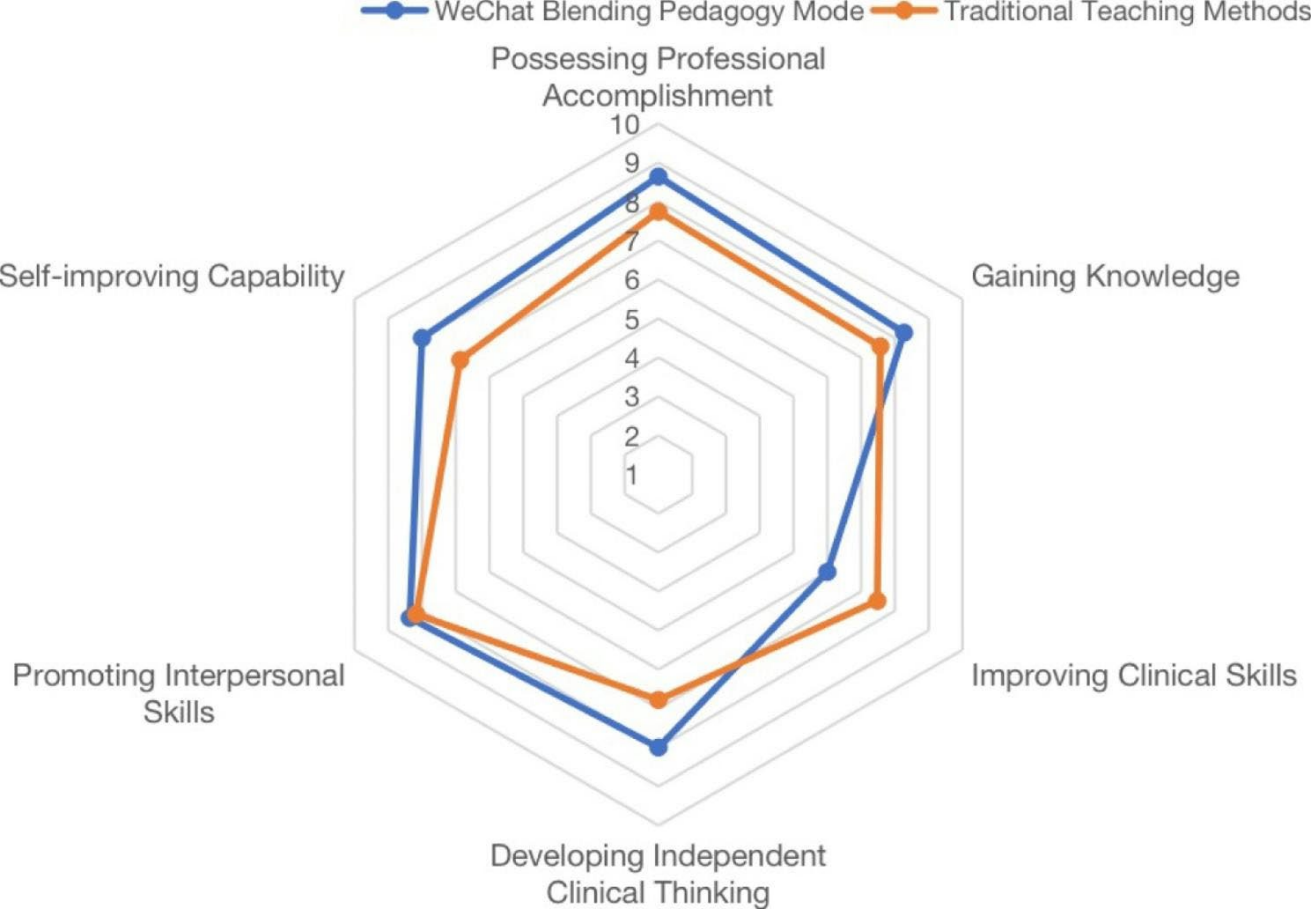
Radar chart of the WeChat blended pedagogy mode and the traditional teaching method based on six dimensions

It is worth noting that in terms of improving clinical skills, the performance of the WeChat blended pedagogy mode was not satisfactory (average score 6.00 < 7.48, *P* = 0.002) (shown in Table 2). This dimension is the only area in which the WeChat blended pedagogy mode scores worse than those associated with the traditional teaching method, a deficiency which is related to the lack of actual hands-on operations in online teaching (Fig. 2).

### Subjective evaluations of the wechat blended pedagogy mode

To investigate intern doctors’ perceptions of the ongoing WeChat blended pedagogy mode, we employed a questionnaire that was measured on a 5-point Likert scale. The questionnaire consisted of 10 questions. Question 1 investigated students’ overall evaluations of the WeChat blended pedagogy mode, while questions 2 to 7 corresponded to the six abilities of students that were tested by the departmental rotation examination. The departmental rotation examination is an objective evaluation of students by teachers, while the questionnaire is a subjective evaluation of students’ personal performance in the WeChat blended pedagogy mode. Questions 8 and 9 investigated WeChat as a teaching platform in terms of convenience and interaction between the teacher and the students, respectively. Question 10 was designed to investigate the time spent by students preparing for the course in the WeChat blended pedagogy mode.

The response rate to the questionnaire was 100% (22/22). The results of the first question indicated that the WeChat blended pedagogy mode had positive effects. Overall satisfaction reached 100% (Table 3). As shown in Table 3, students were satisfied with what they were able to achieve through this new mode of teaching. The majority of students reported that their professional accomplishment, knowledge absorption, independent clinical thinking skills, English reading and literature exploring capacity, and interpersonal skills were improved through the use of the WeChat blended pedagogy mode (in response to Questions 2–3 and 5–7, 64%, 86%, 68%, 64% and 59% of students chose “very large”, while all others chose “large”). Reflecting the problems detected in students’ departmental rotation examination results, more than half of the students’ subjective evaluations noted that the WeChat blended pedagogy mode was less helpful to them with regard to promoting the improvement of their clinical skills (Question 4, 15/22, 68%). A minority of students (Question 8, 3/22, 14%) claimed that it was difficult to make judgements regarding the convenience of WeChat as a teaching platform, and poor interactions with teachers were reported by only 2 students (Question 9, 2/22, 9%). Due to the need to prepare the materials used in discussions in the context of problem/case-based learning and to read the literature materials assigned by the teachers before class, 41% of the students (9/22) believed that the WeChat blended pedagogy mode was time-consuming, 32% of the students (7/22) noted that it was difficult to evaluate the amount of time this preparation consumed, and only 6 students (n = 22, 27%) believed that the time thus required was not excessive.

**Table 3:** The results of the anonymous subjective questionnaire

Question	Response on Likert Scale (n = 22)				
	1	2	3	4	5
1	0 (0%)	0 (0%)	0 (0%)	7 (32%)	15 (68%)
2	0 (0%)	0 (0%)	0 (0%)	8 (36%)	14 (64%)
3	0 (0%)	0 (0%)	0 (0%)	3 (14%)	19 (86%)
4	0 (0%)	7 (32%)	8 (36%)	6 (27%)	1 (5%)
5	0 (0%)	0 (0%)	0 (0%)	7 (32%)	15 (68%)
6	0 (0%)	0 (0%)	0 (0%)	8 (36%)	14 (64%)
7	0 (0%)	0 (0%)	0 (0%)	9 (41%)	13 (59%)
8	0 (0%)	0 (0%)	3 (14%)	8 (36%)	11 (50%)
9	0 (0%)	0 (0%)	2 (9%)	8 (36%)	12 (55%)
10	0 (0%)	9 (41%)	7 (32%)	6 (27%)	0 (0%)

## Discussion

The WeChat blended pedagogy mode was based on the WeChat platform and combined the virtues of PBL, CBL and paper review teaching modes. This approach to teaching was designed to achieve the goal of promoting effective, high-quality learning in the context of undergraduate paediatric orthopaedics internships that could reach or even exceed the teaching quality of offline teaching. In our study, the total average score of students who participated in the WeChat blended pedagogy mode was higher than that of students who participated in the traditional teaching method. The majority of students reported that their professional accomplishment, knowledge absorption, independent clinical thinking skills, English reading and literature exploring capacity, and interpersonal skills were improved by the WeChat blended pedagogy mode and claimed that WeChat was a convenient and effective platform that could serve as a new method of teaching to maintain the continuity of medical education during the COVID-19 pandemic, thus highlighting the acceptability and effectiveness of WeChat as a platform for blended problem/case-based learning and paper review methods in the context of undergraduate paediatric orthopaedics internships.

Due to the COVID-19 outbreak, online teaching, which is not constrained by physical and temporal limitations, replaced traditional offline teaching in many countries [26]. The findings of this study illustrate a method through which online blended pedagogy teaching can be facilitated via WeChat. Compared with the use of similar social media platforms such as WhatsApp [27] and Twitter [28], WeChat offers some special functions that are particularly well adapted for online education, including simple operation, easy obtainability by Chinese students, video conferencing and document sharing [19, 22, 29–31]. Therefore, it can be used to facilitate student-student and student-teacher interactions and communications, promote participation in group discussions and thus improved the learning efficiency and learning quality.

In contrast to an in-person clinical visit, online education faces particular challenges due to dependence of clinical medical education on hands-on training [32]. All trainees at the Qilu Hospital of Shandong University had transitioned to the online mode of teaching in 2020, which greatly reduced their real sense of medical practice and subjective initiative. To simulate real clinical scenes more accurately, we combined PBL and CBL teaching methods, and standardized patients were included in the teaching activities to simulate the real patient treatment process in this study. Previous studies have shown that attempts have been made to implement PBL, CBL or standardized patient teaching models in the context of various college and university majors. For instance, PBL allows students to learn independent problem-solving skills and to obtain fundamental knowledge, even clinical knowledge [17, 33]. By focusing on the preparation of clinical case materials, CBL can stimulate students’ desire to learn and help them develop skills in independent thinking and analysis [17, 34, 35]. Meanwhile, standardized patients play a major role in teaching medical students and helping them to acquire clinical experience by interacting with standardized patients, including by training trainee doctors’ inquiry skills, improving their ability to collect medical history, promoting theie interpersonal skills and cultivating in them a sense of mission and responsibility [21, 36, 37]. However, no study has focused on a combination of PBL, CBL and standardized patient teaching in clinical medicine. According to the analysis of students’ departmental rotation examination scores and self-perceived competence as measured by the questionnaire in our study, we confirmed that these three methods could complement and reinforce each other.

However, it is worth noting that in terms of improving students’ clinical skills, the performance of the WeChat blended pedagogy mode was not satisfactory. For example, when asked to demonstrate external fixation using a plaster bandage on a supracondylar fracture of the humerus during the examination, the performance of many trainee doctors was poor in terms of proficiency, completion time and aesthetics. This dimension was the only context in which the online mode was worse than the traditional teaching method according to the results of our study. This deficiency may be related to the fact that online teaching lacks practical hands-on operations. This finding is consistent with the research of Khalil R et al. [38], who claimed that clerking patients cannot be replaced by online learning as “clinical experience and human interaction are extremely important for the practice of medicine” and online learning cannot completely replace live in-person live sessions. A similar situation occurred with respect to online dental teaching practices [39]. Despite this finding, efforts have been made to overcome these disadvantages. Stephan et al. [40] integrated immersive virtual reality into anatomy teaching by focusing on the reconstruction of cerebral anatomy images, which led to better engagement, more enjoyment, greater utility, and stronger learner motivation. A systematic review and meta-analysis of 11 studies and 715 participants suggested that basic surgical skills can be taught as effectively through online video-based education as through conventional teaching methods [41]. All these attempts have aimed to bring the online teaching model closer to clinical practice to ensure that online teaching is no less effective than offline teaching.

Moreover, to promote the literature retrieval ability of intern doctors more effectively, expand their understanding of the frontier progress of related diseases, and improve their participation in the discussions associated with the teaching process in the context of problem/case-based learning, a paper review method was added to this study. Although these purposes were achieved to a certain extent, based on the results of intern doctors’ questionnaires, this approach also increased students’ preparation time before class. The reasons for the time-consuming nature of this approach may include the following. First, both CBL and PBL are reported to entail a heavier workload for students to be fully prepared, which is time-consuming [10, 42]. Second, the level of English proficiency exhibited by intern doctors varies, so some interns may spend too much time reading English literature. The paper review method was used only to teach master’s or doctoral students [20], and this case was the first time that this approach was applied to the task of teaching intern doctors via problem/case-based learning. However, this attempt was successful; this approach improved the intern doctors’ English literature reading ability and promoted their self-improving capability. Finally, in the absence of actual patients, intern doctors’ understanding of paediatric orthopaedic diseases is underdeveloped, which requires a great deal of time to master.

However, our research faced several limitations. First, in view of the discipline characteristics of pediatric surgery and many other factors, the number of undergraduates who chose to rotate pediatric surgery was relatively small, resulting in a small sample size in this study. Second, the evaluation method in the research was relatively subjective. In China, students’ learning performance was mainly measured by examination scores. What’s more, since there was no blind method in our study, some analysis bias is unavoidable. We acknowledge that teachers’ assessments of intern doctors may have been influenced by subjective factors, including improved interpersonal bonds that formed over time. Further research will combine with entrustable professional activities (EPAs), an emerging clinical teaching assessment method, to track and assess students’ ability to perform clinical practice remotely, while enhancing clinical assessment of professional competence in pediatric surgery. Third, influenced by government policy, this study was not a randomized controlled study, but a retrospective control group was selected to be included in the study. Fourth, our study analysed data drawn from only one clinical department within our institution, and these results may have exhibit differences outside this context. At last, this study lacked feedback concerning long-term online teaching practices. In the future, we will conduct an experiment with multiple central randomized trials, a large sample size, and long-term follow-up.

## Conclusions

Online teaching is the main teaching method used in China during the COVID-19 epidemic, and paediatric orthopaedics is a practical discipline that is difficult to master. We integrated PBL, CBL and paper review teaching modes into the teaching of paediatric orthopaedic intern doctors via the WeChat platform, developed the WeChat blended pedagogy mode and verified its feasibility and effectiveness in terms of teaching practice. According to the results of our study, the WeChat blended pedagogy mode can improve many skills of these intern doctors beyond the level attained by the traditional teaching method, including their professional accomplishment, knowledge absorption, independent clinical thinking skills, English reading and literature exploring capacity, and interpersonal skills. However, further improvement and refinement is necessary in terms of clinical skills. In addition, some intern doctors claimed that the WeChat blended pedagogy mode took too much time before class, which may be related to their personal level of English skills; thus, long-term observation and practice are required to make the study more robust and produce more grounded assessments.

## Electronic supplementary material

Below is the link to the electronic supplementary material.

Supplementary Material 1

Supplementary Material 2

## Data Availability

The original data were deposited into the Mendeley Data, V1(https://datamendeley.com/datasets. ) with DOI: 10.17632/6n53cw9ng3.1.

## List of abbreviations

COVID-19: Coronavirus Disease 2019
CBL: Case-Based Learning
PBL: Problem-Based Learning
