# Supplementary material for: WeChat as a platform for blending problem/case-based learning and paper review methods in undergraduate paediatric orthopaedics internships: a feasibility and effectiveness study
Source: BMC Med Educ. 2023 May 8;23:322. doi: 10.1186/s12909-023-04269-2 (PMC10166020; doi:10.1186/s12909-023-04269-2)
Supplement: Supplementary file 1 — Supplementary Material 1 [file 12909_2023_4269_MOESM1_ESM.docx]

**Table S1** Departmental Rotation Examination Score Sheet

| Items | Detailed Grading Rules | Scores |
| --- | --- | --- |
| Possessing Professional Accomplishment | Intern doctors are compassionate, responsible and altruistic, and fulfill the "patient-centered" medical philosophy. | ☐10 ☐8 ☐6 ☐4 ☐2 |
| Gaining Knowledge | Intern doctors have evidence-based medicine thinking. according to professional guidelines, following the best evidence, they can combine with clinical experience and patient needs, balance, select and implement rational diagnosis and treatment decisions. | ☐10 ☐8 ☐6 ☐4 ☐2 |
| Improving Clinical Skills | Intern doctors master the clinical skills required by the specialty and have the ability to practice medicine independently in this specialty. | ☐10 ☐8 ☐6 ☐4 ☐2 |
| Developing Independent Clinical Thinking | With the guarantee of patients' medical safety as the core, intern doctors can use their professional abilities to develop individualized diagnosis and treatment plans and provide effective and appropriate medical care services. | ☐10 ☐8 ☐6 ☐4 ☐2 |
| Promoting Interpersonal Skills | Intern doctors have humanistic feelings of clinical communication ability, to establish mutual trust and harmonious doctor-patient relationship, to solve practical clinical problems. | ☐10 ☐8 ☐6 ☐4 ☐2 |
| Self-improving Capability | With the concept of independent learning and lifelong learning, intern doctors take the initiative to use various academic resources to continuously track medical progress, update medical knowledge and concepts, and carry out or participate in scientific research based on clinical problems and needs. | ☐10 ☐8 ☐6 ☐4 ☐2 |
