# Supplementary material for: WeChat as a platform for blending problem/case-based learning and paper review methods in undergraduate paediatric orthopaedics internships: a feasibility and effectiveness study
Source: BMC Med Educ. 2023 May 8;23:322. doi: 10.1186/s12909-023-04269-2 (PMC10166020; doi:10.1186/s12909-023-04269-2)
Supplement: Supplementary file 2 — Supplementary Material 2 [file 12909_2023_4269_MOESM2_ESM.docx]

**Table S2** Anonymous Subjective Questionnaire (10 questions)

| Question | Options |
| --- | --- |
| 1. Your overall evaluation about these courses: | ☐Dissatisfied ☐Moderately dissatisfied  ☐Difficult to judge ☐Satisfied ☐Very satisfied |
| 1. This teaching mode is conducive to the improvement of doctors' compassionate, responsible and altruistic professionalism, and fulfill the philosophy of patient-centered medicine: | ☐Small ☐Moderate, indifferent  ☐Difficult to judge ☐Large ☐Very large |
| 1. This teaching mode helped you to understand and use the basic knowledge: | ☐Small ☐Moderate, indifferent  ☐Difficult to judge ☐Large ☐Very large |
| 4. In relation to improving your clinical skills in this teaching mode, your learning was: | ☐Small ☐Moderate, indifferent  ☐Difficult to judge ☐Large ☐Very large |
| 5. In relation to training your clinical thinking ability in this teaching mode, your learning was: | ☐Small ☐Moderate, indifferent  ☐Difficult to judge ☐Large ☐Very large |
| 6. In relation to improving English reading and literature exploring capacity in this teaching mode, your learning was: | ☐Small ☐Moderate, indifferent  ☐Difficult to judge ☐Large ☐Very large |
| 7. In relation to cultivating capability of patient-doctor communication in this teaching mode, your learning was: | ☐Small ☐Moderate, indifferent  ☐Difficult to judge ☐Large ☐Very large |
| 8. It is very convenient to use WeChat as a teaching platform: | ☐Very inconvenient ☐Inconvenient ☐Difficult to judge ☐Convenient ☐Very convenient |
| 9. The interaction between the teacher and the students during the mode: | ☐No interaction ☐Uncommon ☐Moderate, indifferent ☐Poor interaction ☐Common |
| 10. Compared with the traditional model of lecture, does this teaching mode consumed more free time to prepared before class to learning: | ☐Super much ☐Much ☐Difficult to judge  ☐Indifferent, moderate ☐Little |
